# Supplementary material for: Quantifying the impact of ecological memory on the dynamics of interacting communities
Source: PLoS Comput Biol. 2022 Jun 3;18(6):e1009396. doi: 10.1371/journal.pcbi.1009396 (PMC9200327; doi:10.1371/journal.pcbi.1009396)
Supplement: S1 Appendix — (PDF) [file pcbi.1009396.s001.pdf]

S1 Appendix: Methodological details for Figs 5C and S1

Fig 5C: Ternary plots allow representing the state of a 3-species or 3-group system by a single dot and therefore are a convenient way to display the outcome of many simulations at a time. In Fig 5C, each ternary plot shows the stable state distribution of the group relative abundances obtained for 50 different simulations, each represented by a dot of the color of the dominant group. We detail below how we computed the position of each dot in a triangle (Fig A1). Let us write B, G and R the average stable state

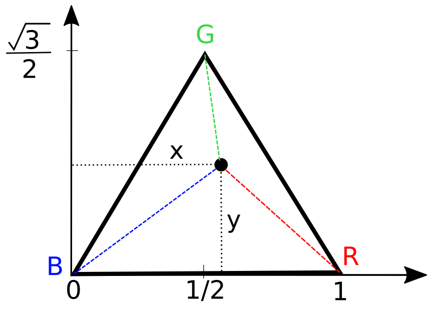

Fig A1. Triangle coordinates.

relative abundances of the species in the blue, green and red groups, that is $R = \dfrac{\sum_{i=1}^{5} R_i(end)}{\sum_i (R_i + B_i + G_i)(end)}$ (and similarly for B and G), where $Z_i(end)$ denotes the abundance of species i in group Z at the end of the simulation. Let us consider an equilateral triangle in which each vertex corresponds to the complete dominance of one group of species, as shown in Fig A1. Thus, a point (dot) close to the middle of the triangle indicates a state of the system characterized by relatively even species abundances. If $B = 1$ (100%) is placed at $(x, y) = (0, 0)$ and $R = 1$ (100%) at $(1, 0)$, then $G = 1$ (100%) is at $(\frac{1}{2}, \frac{\sqrt{3}}{2})$, and any triplet (B, R, G) will be at $(x, y) = \left(\frac{1}{2}(2R + G), \frac{\sqrt{3}}{2}G\right)$. These Cartesian coordinates provide a way to map any triplet of group relative abundances to a unique location on the triangle.

S1 Fig: Here, we randomly generated an interaction matrix $\mathbf{K}$ without predefined structure between $N = 15$ species. Specifically, we set $n = 4$ and $K_{ij} = 1 - e^{-5z}$, where z is a randomly generated number from a uniform distribution between 0 and 1. We generated 10 communities, each with a random vector of growth rates generated as $b_i \sim \mathcal{N}(1, 0.0025)$, $\forall i$. We used the same interaction matrix for all 10 communities, and death rates $k_i = 2$, $\forall i$. For each community, we set the initial values for species abundances X_i at one of the equilibrium points of the system (randomly chosen). To compute the dissimilarity of the community between times t_r and t_p, we used the Bray-Curtis distance, computed as $BC(t_r, t_p) = \dfrac{\sum_{i=r}^{N} |X_i(t_r) - X_i(t_p)|}{\sum_{i=1}^{N} X_i(t_r) + X_i(t_p)}$.
