# Supplementary material for: Quantifying the impact of ecological memory on the dynamics of interacting communities
Source: PLoS Comput Biol. 2022 Jun 3;18(6):e1009396. doi: 10.1371/journal.pcbi.1009396 (PMC9200327; doi:10.1371/journal.pcbi.1009396)
Supplement: S2 Appendix — (PDF) [file pcbi.1009396.s002.pdf]

S2 Appendix: Numerical simulations

In the following, we describe the numerical algorithm that we used to solve fractional-order differential equation systems.

Adams methods provide commonly used numerical solutions for ordinary differential equations, involving implicit (Adams-Moulton) and explicit (Adams-Bashforth) linear multi-step schemes. We exploited in this paper the predictor-corrector method based on Adams formulae (see [1]).

Given the system equation (3) in the Methods, let us write $\mathbf{X}$ the set of all species abundances, $\boldsymbol{\mu}$ the corresponding vector of derivative orders μ_i, and $\mathbf{F}$ the corresponding matrix function of all $F_i = X_i\left(b_i f_i(\{X_k\}) - k_i X_i\right)$. We can then rewrite the fractional order model (3) in the following matrix form:

$$\mathfrak{D}^{\boldsymbol{\mu}}\mathbf{X} = \mathbf{F}(t, \mathbf{X}), \text{ where } \mathbf{X}(t_0) = \mathbf{X}_0. \tag{i}$$

The initial value problem (i) is equivalent to the Volterra integral equation [2]:

$$\mathbf{X}(t) = \mathbf{X}_0 + \frac{1}{\Gamma(\boldsymbol{\mu})}\int_{t_0}^{t}(t-\tau)^{\boldsymbol{\mu}-1}\mathbf{F}(\tau, \mathbf{X}(\tau))d\tau. \tag{ii}$$

We solved Eq. (ii) using a product integration technique, in which we replaced the function $\mathbf{F}(\tau, \mathbf{X}(\tau))$ with piece-wise interpolating polynomials. For the grid nodes t_j $(j = 0, ..., m)$ with constant step size h $(t_j = t_0 + jh)$, we write $\mathbf{F}_j = \mathbf{F}(t_j, \mathbf{X}_j)$ where $\mathbf{X}_j$ is the numerical approximation to $\mathbf{X}(t_j)$. The product rectangle rule [2] gives an explicit estimation of Eq. (ii) as a predictor:

$$\begin{gathered}\mathbf{X}_m = \mathbf{X}_0 + h^{\boldsymbol{\mu}}\textstyle\sum_{j=0}^{m-1}\mathbf{b}_{m-j-1}\mathbf{F}_j, \\ \mathbf{b}_{m-j-1} = \frac{(m-j)^{\boldsymbol{\mu}}-(m-j-1)^{\boldsymbol{\mu}}}{\Gamma(\boldsymbol{\mu}+1)},\end{gathered} \tag{iii}$$

and the product trapezoidal rule [2] provides an implicit estimation of Eq. (ii) as a corrector:

$$\begin{gathered}\mathbf{X}_m = \mathbf{X}_0 + h^{\boldsymbol{\mu}}\mathbf{c}_m\mathbf{F}_0 + h^{\boldsymbol{\mu}}\textstyle\sum_{j=1}^{m}\mathbf{d}_{m-j}\mathbf{F}_j, \\ \mathbf{c}_m = \frac{(m-1)^{\boldsymbol{\mu}+1}-m^{\boldsymbol{\mu}}(m-\boldsymbol{\mu}-1)}{\Gamma(\boldsymbol{\mu}+2)}, \\ \mathbf{d}_{m-j} = \begin{cases}\frac{1}{\Gamma(\boldsymbol{\mu}+2)}, & \text{if } m-j=0, \\ \frac{(m-j-1)^{\boldsymbol{\mu}+1}-2(m-j)^{\boldsymbol{\mu}+1}+(m-j+1)^{\boldsymbol{\mu}+1}}{\Gamma(\boldsymbol{\mu}+2)}, & \text{if } m-j=1,2,...\end{cases}\end{gathered} \tag{iv}$$

The last term of the sum in the corrector equation (iv), $\mathbf{F}(t_m, \mathbf{X}_m)$, is obtained by an approximation of $\mathbf{X}_m$ in the predictor equation (iii). This method is called FracPECE (Fractional Predict Evaluate Correct Evaluate) [2]. Because its standard implementation was not sufficient considering the stiffness of the equation, we improved its accuracy via an advanced convolution quadrature and Fast Fourier Transform [1], and via multiple applications of the corrector step [3] when required. Specifically, we used several corrector iterations when the difference between two consecutive iterations was larger than the desired tolerance of 10^{-6}.

Note that since the model with fractional order derivatives (3) includes the standard model (1) as a particular case (namely, for integer derivative order), the numerical approximations (iii) and (iv) are also solutions to equation (1). The explicit solution (iii)–or an assessment of the implicit solution (iv)–shows how memory influences the fundamental system dynamics through the dependence on μ.

References

1. Garrappa R. Numerical Solution of Fractional Differential Equations: A Survey and a Software Tutorial. Mathematics. 2018;6(2). doi:10.3390/math6020016.

2. Diethelm K, Ford NJ, Freed AD. A predictor-corrector approach for the numerical solution of fractional differential equations. Nonlinear Dyn. 2002;29(1-4):3–22. doi:10.1023/A:1016592219341.

3. Diethelm K, Ford NJ, Freed AD. Detailed error analysis for a fractional Adams method. Numer Algorithms. 2004;36(1):31–52. doi:10.1023/B:NUMA.0000027736.85078.be.
