# Supplementary material for: Quantifying the impact of ecological memory on the dynamics of interacting communities
Source: PLoS Comput Biol. 2022 Jun 3;18(6):e1009396. doi: 10.1371/journal.pcbi.1009396 (PMC9200327; doi:10.1371/journal.pcbi.1009396)
Supplement: S1 Table — (PDF) [file pcbi.1009396.s003.pdf]

Table S1. Exact model specifications for the 2, 3, and 15-species Gonze model (equation 1 in Methods).

**Figure**				**Gonze model (1)**								
Commensurate	X_0			K_{ij}	n	k_i	μ			b		
	B	R	G	$\forall i \neq j$		$\forall i$	B=R=G			B	R	G
**2A**<br>**2B**<br>**3A**<br>**3B**	0.99	0.01	0.01	0.1	2	1	1 & 0.9<br>1 & 0.9<br>1 & 0.96 & 0.9<br>1 & 0.96 & 0.9			Pulse1<br>Pulse2<br>Pulse3<br>Periodic	0.95	Pulse1<br>Pulse2<br>1.05<br>1.05
**4B-C**	1/3	1/3	1/3	0.1	2	1	1<br>0.9157959<br>0.9157954<br>0.9157952<br>0.9<br>0.7<br>0.6			Stochastic		
Incommensurate							B	R	G			
**S1**	Equilibrium points			Random interactions	4	2	No Specified Groups μ_i, $\forall i = 1$ (or 0.7)			$\mathcal{N}(1, 0.0025)$ with a pulse		
**5**	$Uniform(0, 0.1)$			Predefined interactions	2	1	1<br>0.6<br>0.851841<br>0.851840<br>0.8	1	1	$\mathcal{N}(1, 0.01)$		
**S2**							permutation of 1, 0.9, 0.6					
**S3A**	0.99	0.01	0.01	0.1	2	1	1<br>1<br>1	1<br>1<br>1	1<br>0.90895<br>0.90893	Pulse4	0.95	1.05
**S3B**	1/3	1/3	1/3	0.1	2	1	1<br>1<br>1	1<br>1<br>0.9	1<br>0.8<br>1	Stochastic		
**S4A**<br>**S4B** & **6**	[0.005,0.05]	0.1&0.3	1&0.1	0.1	2	1	1<br>0.6	1<br>0.6	1<br>1	4	0.95	1.05

**2-Species**	X_0		K_{ij}	n	k_i	μ		b		Convergence interval
	B	R	$\forall i \neq j$		$\forall i$	B	R	B	R	
**S5A** & **B**	0.8 & 0.9	0.2 & 0.15	0.1	2	1	[0.6,1]	[0.6,1]	1	2	-
**S9**	0.9	0.2				[0.84,1]	[0.84,1]			5e-3
**S7**	Equilibrium points					[0.9,1]	[0.9,1]	Pulse5		-
**S8**										0.02 & 1e-6

Pulse1: $b_B(t) = 0.5$ and $b_G(t) = 2$ if $20 \leq t < 60$, otherwise $b_B(t) = 1$ and $b_G(t) = 1.05$.
Pulse2: $b_B(t) = 0.5$ and $b_G(t) = 2.2$ if $20 \leq t < 60$, otherwise $b_B(t) = 1$ and $b_G(t) = 1.05$.
Pulse3: $b_B(t) = 0.2$ if $60 \leq t < 100$, $b_B(t) = 4.5$ if $200 \leq t < 330$, otherwise $b_B(t) = 1$.
Periodic: $b_B(t) = 1$ if $20(2m-2) \leq t < 20(2m-1)$, $b_B(t) = 0.2$ if $20(4m-3) \leq t < 20(4m-2)$, $b_B(t) = 4.5$ if $20(4m-1) \leq t < 20(4m)$ where $m \in \mathbb{N}$.
Stochastic: The growth rates of these panels are generated by mean-reverting the Ornstein-Uhlenbeck Process described by the stochastic equation $db_t = \theta(\phi - b_t)dt + \sigma dW_t$.
Random interactions: $K_{ij} = 1 - e^{-5z}$, where z is randomly generated from a uniform distribution between 0 and 1.
Predefined interactions: $K_{ij} \sim 1 + \mathcal{N}(0, 0.01)$ for species i and j in the same group (intra-group interactions K_{BB}, K_{RR}, K_{GG}), and $K_{ij} \sim 0.5 + \mathcal{N}(0, 0.01)$ for species i and j in different groups (inter-group interactions).
Pulse4: $b_B(t) = 0.2$ if $60 \leq t < 100$, $b_B(t) = 4.5$ if $400 \leq t < 530$, otherwise $b_B(t) = 1$.
Pulse5: $b_R(t) = 2$ and $b_B(t) = b_B(t) + p$ if $50 \leq t < 100$, otherwise $b_B(t) = 1$, where p is a positive value.
