# Supplementary material for: Quantifying the impact of ecological memory on the dynamics of interacting communities
Source: PLoS Comput Biol. 2022 Jun 3;18(6):e1009396. doi: 10.1371/journal.pcbi.1009396 (PMC9200327; doi:10.1371/journal.pcbi.1009396)
Supplement: S2 Table — (PDF) [file pcbi.1009396.s004.pdf]

Table S2. Exact model specifications for the 2-species model given by equation 2 in Methods, and for the logistic growth curve in Fig 8.

**Logistic growth curve**		X_0		κ		μ		b			
**Fig 8**		0.1		1		[0.6:1]		1			
**Model (2)**	X_0		K_{ij}				μ		b		Convergence
**Figure**	BU	BT	BUBU	BUBT	BTBU	BTBT	BU	BT	BU	BT	interval
**7A & B**	0.0083 & 0.0113	0.0117	-0.9059	-0.9377	-0.9720	-0.9597	[0.6,1]	[0.6,1]	0.599	0.626	-
**S10**	0.0083	0.0117					[0.84,1]	[0.84,1]			5e-3
**S6**	Equilibrium points						[0.9,1]	[0.9,1]	Pulse6		-
**7C-F**							[0.9,1]	[0.9,1]			0.02 & 7e-4
	CH	ER	CHCH	CHER	ERCH	ERER	CH	ER	CH	ER	Convergence interval
**S5E**	0.0002	0.0198	-1.2420	-0.5077	1.1905	-1.3219	[0.6,1]	[0.6,1]	0.468	0.151	-
**S11**							[0.9,1]	[0.9,1]			5e-4
	BT	CH	BTBT	BTCH	CHBT	CHCH	BT	CH	BT	CH	Convergence interval
**S5F**	0.0035	0.0165	-0.9597	-0.0727	-0.5906	-1.2420	[0.6,1]	[0.6,1]	0.626	0.468	-
**S12**							[0.9,1]	[0.9,1]			1e-3

Pulse6: $b_{BT}(t) = 0.626$ and $b_{BU}(t) = b_{BU}(t) + p$ if $50 \leq t < 80$, otherwise $b_{BU}(t) = 0.599$, where p is a positive value.

Logistic growth curve: a well-known model of biological population dynamics, which describes simple exponential growth. $\frac{dX}{dt} = b\left(1 - \frac{X}{\kappa}\right)X$, where X represents the population size, b the growth rate, and κ the carrying capacity.
