# Supplementary material for: Quantifying the impact of ecological memory on the dynamics of interacting communities
Source: PLoS Comput Biol. 2022 Jun 3;18(6):e1009396. doi: 10.1371/journal.pcbi.1009396 (PMC9200327; doi:10.1371/journal.pcbi.1009396)
Supplement: S1 Fig — (PDF) [file pcbi.1009396.s005.pdf]

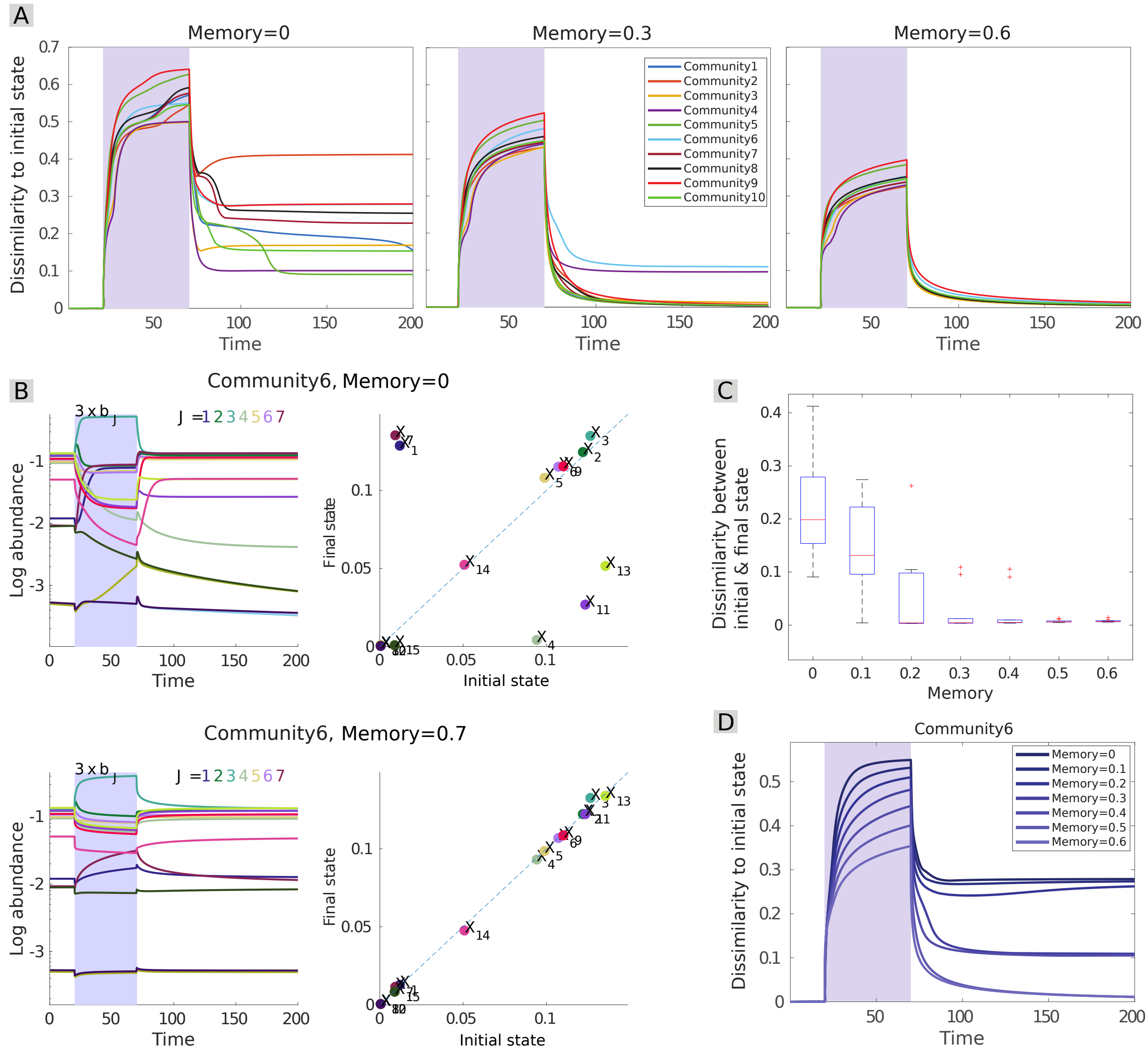

Fig S1. Memory effects preserve the stable state in randomly structured communities. We simulated ten communities of 15 species each with random interaction matrices (see S1 Appendix for details). We applied a similar level of commensurate memory to all ten communities. Every community is initially in a stable state of the system, and a perturbation is imposed by multiplying the growth rates of half of the species ($b_1, ..., b_7$) by 3. The simulation is stopped when the system is close to its new stable state. Although only the effect of commensurate memory is illustrated here, the same outcome can be achieved using incommensurate memory. **(A)** Dissimilarity (Bray-Curtis) to the initial stable state through time for all ten communities, for three different memory strengths. The stronger the memory, the more constrained the community trajectories are, and the more likely they are to revert to their initial stable state eventually. **(B)** Time series for one randomly chosen community, community 6. The pulse perturbations lead the community to an alternative stable state in the absence of memory (top), while adding memory effects allows recovering the original state (bottom). **(C)** Community dissimilarity (Bray-Curtis) between the start and the end of the simulation for all ten communities and different memory levels. Without memory, the pulse perturbation changes the abundances of some of the species and leads to an alternative stable state (*i.e.*, non-zero dissimilarity between start and end). In contrast, all communities recover their pre-perturbation stable state in the presence of memory (*i.e.*, zero dissimilarity). **(D)** Dissimilarity to the initial stable state through time in community 6, for different memory strengths.
