# Supplementary material for: Quantifying the impact of ecological memory on the dynamics of interacting communities
Source: PLoS Comput Biol. 2022 Jun 3;18(6):e1009396. doi: 10.1371/journal.pcbi.1009396 (PMC9200327; doi:10.1371/journal.pcbi.1009396)
Supplement: S2 Fig — (PDF) [file pcbi.1009396.s006.pdf]

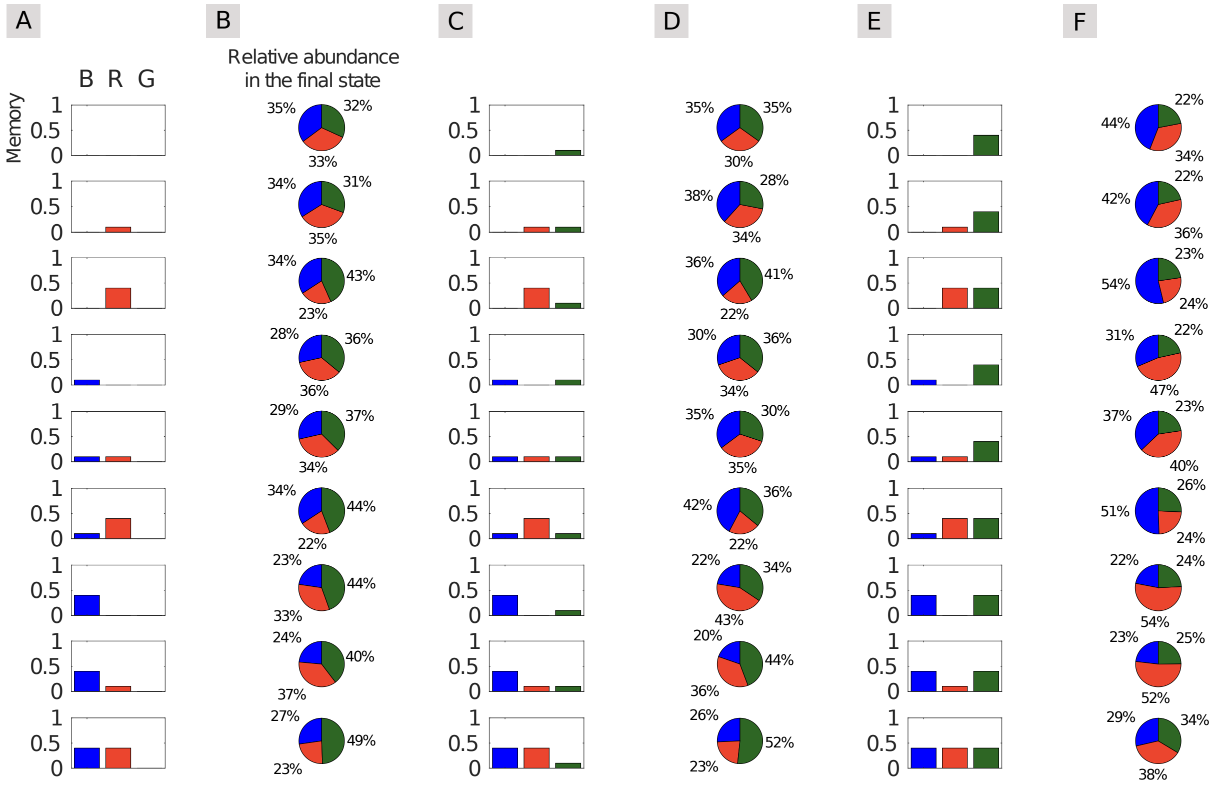

Fig S2. Memory in a group of species decreases their relative abundance. Same as Fig 5C, for varying incommensurate memory values in the three species groups. For each of the 27 memory configurations represented in columns A, C and E (*i.e.*, varying memory strengths for species groups blue, red and green), the same model as in Fig 5 was simulated 50 times with random initial conditions. Columns B, D and E show the corresponding outcomes, represented as the mean relative abundance achieved by each species group in the final state across the 50 simulations. The columns are ordered by increasing strength of memory in the green species group (from left to right), and the rows by increasing strength of memory in the blue species group (from top to bottom). For each of the three groups, increasing the strength of their memory leads to decreasing their relative abundance in the final state.
