# Supplementary material for: Quantifying the impact of ecological memory on the dynamics of interacting communities
Source: PLoS Comput Biol. 2022 Jun 3;18(6):e1009396. doi: 10.1371/journal.pcbi.1009396 (PMC9200327; doi:10.1371/journal.pcbi.1009396)
Supplement: S3 Fig — (PDF) [file pcbi.1009396.s007.pdf]

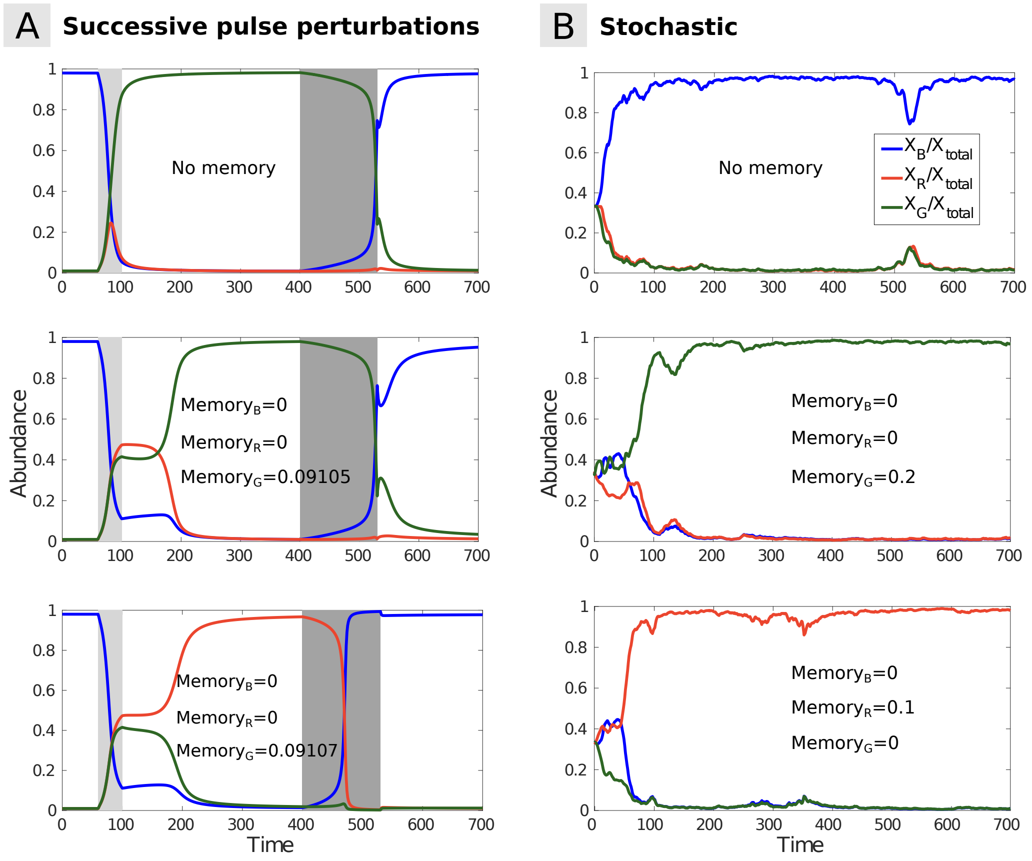

Fig S3. Impact of incommensurate memory in the presence of perturbation. (A) Same as Fig 3A-B but with incommensurate memory: memory is applied to the green species with increasing strength from top to bottom, while the blue and red species remain memoryless. Around a particular memory strength (0.09106), the system changes behavior: when memory in the green species is between 0 and 0.09105, the first perturbation leads to the green species achieving dominance, whereas between 0.09107 and 1, it leads to the red species achieving dominance. **(B)** Same as Fig 4 but with incommensurate memory. In the absence of memory, the blue species is dominant. However, when imposing sufficient memory on the green or red species, they respectively become dominant in the stable state.
