# Supplementary material for: Quantifying the impact of ecological memory on the dynamics of interacting communities
Source: PLoS Comput Biol. 2022 Jun 3;18(6):e1009396. doi: 10.1371/journal.pcbi.1009396 (PMC9200327; doi:10.1371/journal.pcbi.1009396)
Supplement: S4 Fig — (PDF) [file pcbi.1009396.s008.pdf]

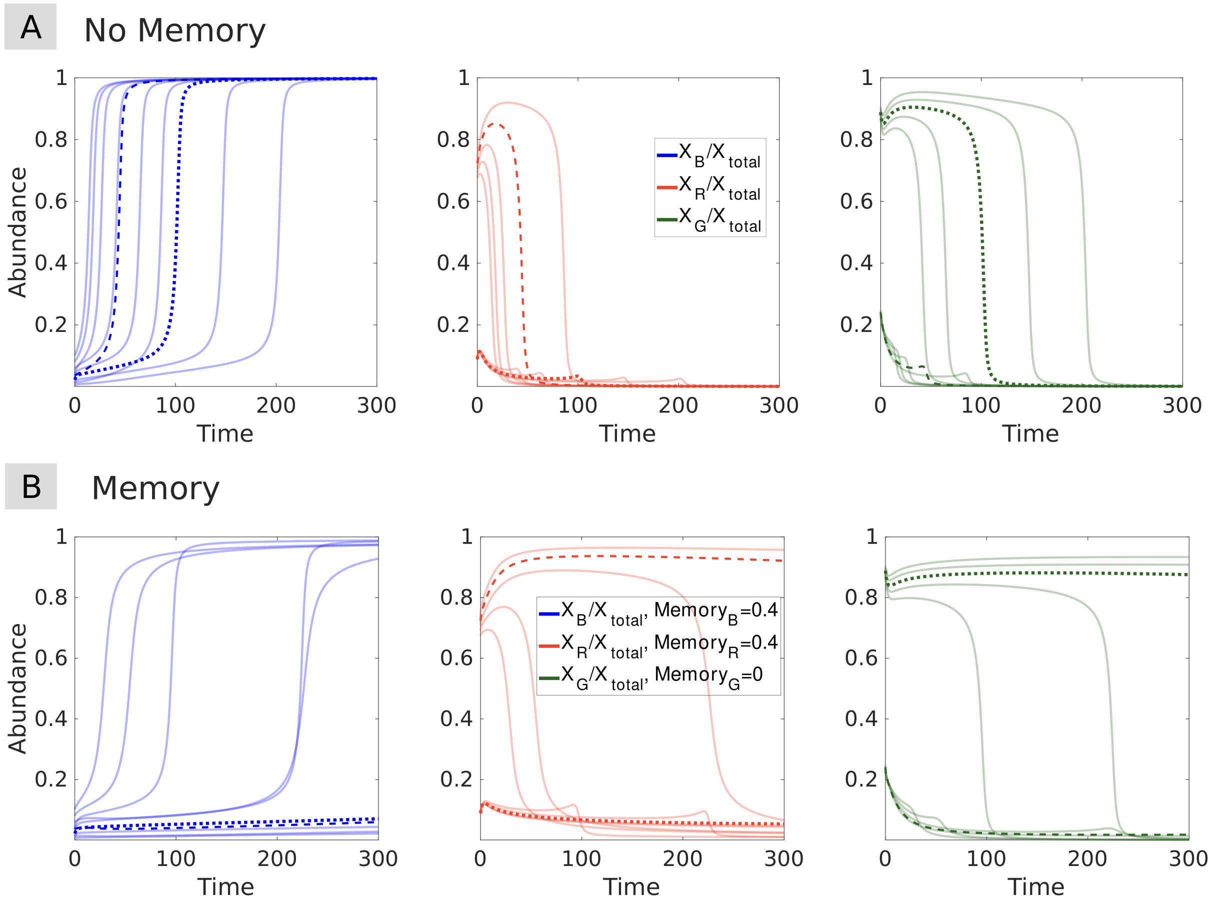

Fig S4. Memory can induce long transient dynamics even in the absence of multistability. Three-species community model converging to a single stable state irrespective of initial conditions, but close enough to the tristable region in the model's parameter space that the introduction of memory induces alternative long transient states (see Fig 6). In each row, the three panels show the relative abundances of the blue, red and green species along time for the same 10 simulations starting from 10 different initial conditions (*i.e.*, sets of initial abundances). **(A)** In the absence of memory, the dynamics quickly converges to the stable state irrespective of initial conditions. **(B)** In the presence of memory, the dynamics may, depending on the initial conditions, remain stuck in alternative transient states far from the stable state (although it would eventually converge to the stable state should the simulation be run long enough). The dashed and dotted lines indicate the initial abundance thresholds that separate from each other the stable state and the two alternative transient regimes, each corresponding to the dominance of a different species. The same initial conditions are also indicated by dashed and dotted lines in (A)
