# Supplementary material for: Quantifying the impact of ecological memory on the dynamics of interacting communities
Source: PLoS Comput Biol. 2022 Jun 3;18(6):e1009396. doi: 10.1371/journal.pcbi.1009396 (PMC9200327; doi:10.1371/journal.pcbi.1009396)
Supplement: S5 Fig — (PDF) [file pcbi.1009396.s009.pdf]

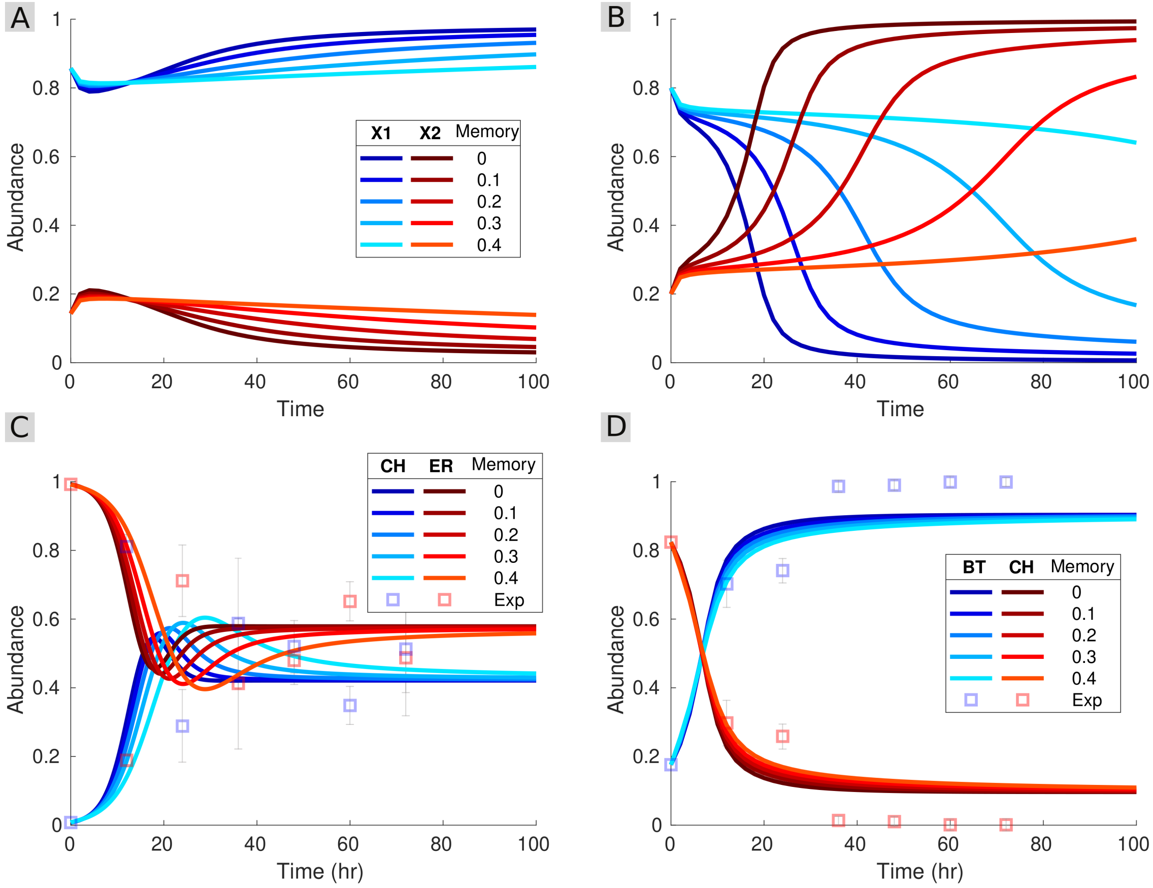

Fig S5. Memory effects on dynamics for different two-species community types. Impact of memory on the dynamics of the different communities considered in the "Empirically parameterized model" section of the results, with a range of memory values for each panel. **(A-B)** Two-species version of the multistable (here bistable) community model from Gonze model (described by equation (1) in the Methods). (A) and (B) show a set of initial abundances leading to one or the other stable state (blue- or red-dominated). **(C)** Monostable community exhibiting coexistence between *Eubacterium rectale* (ER) and *Clostridium hiranonis* (CH) in the stable state. **(D)** Monostable community exhibiting dominance of *Bacteroides thetaiotaomicron* (BT) over *Clostridium hiranonis* (CH) in the stable state. The models for panels (C) and (D) are described by equation (2) in the Methods and the corresponding parameter values, inferred from experimental data by [1], are detailed in Table in S2 Table. Panels (C) and (D) show community dynamics for the same initial conditions as one of the empirical time series used to fit the model. The original experimental data are indicated by squares and (truncated) error bars centered on the squares, representing the mean and standard deviation across at least 3 biological replicates, respectively.

References

[1] Ophelia S Venturelli et al. "Deciphering microbial interactions in synthetic human gut microbiome communities". In: *Mol. Syst. Biol.* 14.6 (2018), e8157. DOI: `https://doi.org/10.15252/msb.20178157`. URL: `https://www.embopress.org/doi/abs/10.15252/msb.20178157`.
