# Supplementary material for: Quantifying the impact of ecological memory on the dynamics of interacting communities
Source: PLoS Comput Biol. 2022 Jun 3;18(6):e1009396. doi: 10.1371/journal.pcbi.1009396 (PMC9200327; doi:10.1371/journal.pcbi.1009396)
Supplement: S6 Fig — (PDF) [file pcbi.1009396.s010.pdf]

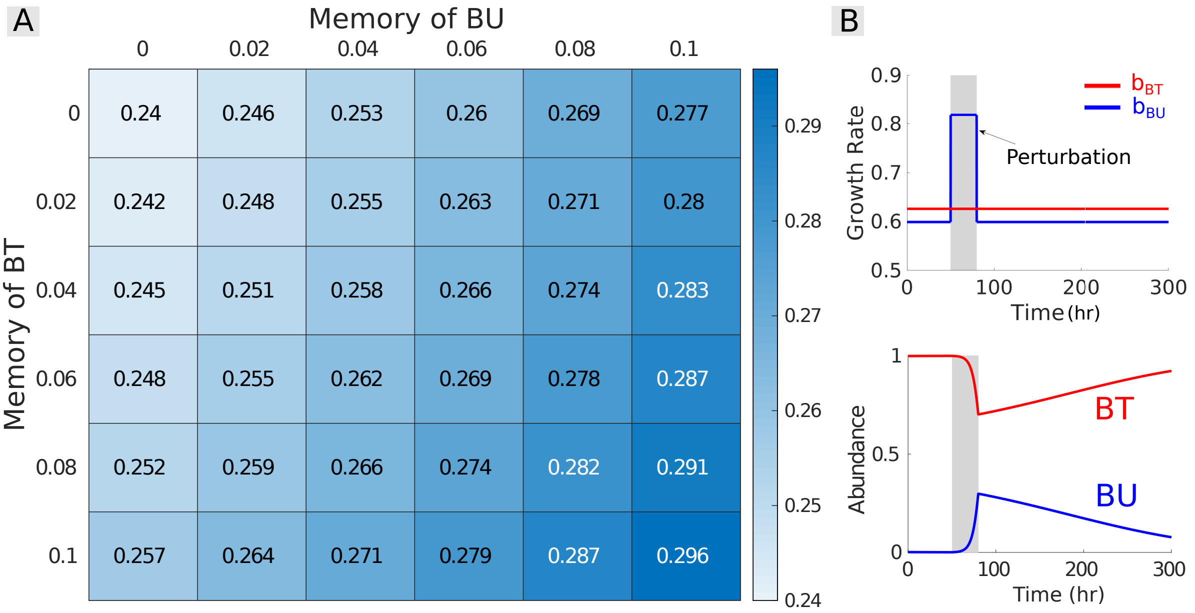

Fig S6. Impact of memory on resistance to a pulse perturbation in a two-species community exhibiting bistability between dominance of *Bacteroides uniformis* (BU) and *Bacteroides thetaiotaomicron* (BT). (A) Both color and matrix entries indicate the strongest pulse perturbation for which the community recovers to its initial stable state, as a function of memory strength in BU and BT. As in S7 Fig, increasing memory in either species increases system resistance in a similar way. **(B)** For each matrix entry in (A), a pulse perturbation is applied to the growth rate of BU (top panel), which temporarily displaces the community away from its original stable state dominated by BT (bottom panel). The strength of the perturbation is defined as the value taken by BU growth rate during the pulse.
