# Supplementary material for: Quantifying the impact of ecological memory on the dynamics of interacting communities
Source: PLoS Comput Biol. 2022 Jun 3;18(6):e1009396. doi: 10.1371/journal.pcbi.1009396 (PMC9200327; doi:10.1371/journal.pcbi.1009396)
Supplement: S7 Fig — (PDF) [file pcbi.1009396.s011.pdf]

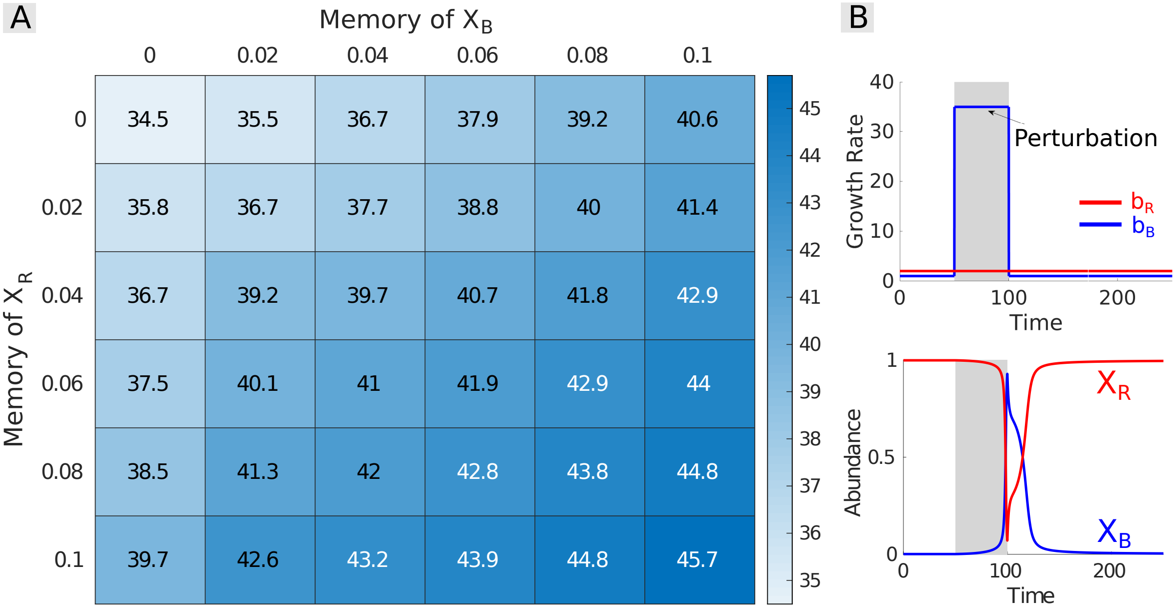

Fig S7. Impact of memory on resistance to a pulse perturbation in the two-species version of Gonze multistable model. (A) Both color and matrix entries indicate the strongest pulse perturbation for which the community recovers to its initial stable state, as a function of memory strength in the blue and red species (abundances X_B and X_R, respectively). Increasing memory in either species increases system resistance in a similar way. **(B)** For each matrix entry in (A), a pulse perturbation is applied to the growth rate of the blue species (top panel), which temporarily displaces the community away from its original stable state dominated by the red species (bottom panel). The strength of the perturbation is defined as the value taken by the blue species growth rate during the pulse.
