# Supplementary material for: Quantifying the impact of ecological memory on the dynamics of interacting communities
Source: PLoS Comput Biol. 2022 Jun 3;18(6):e1009396. doi: 10.1371/journal.pcbi.1009396 (PMC9200327; doi:10.1371/journal.pcbi.1009396)
Supplement: S8 Fig — (PDF) [file pcbi.1009396.s012.pdf]

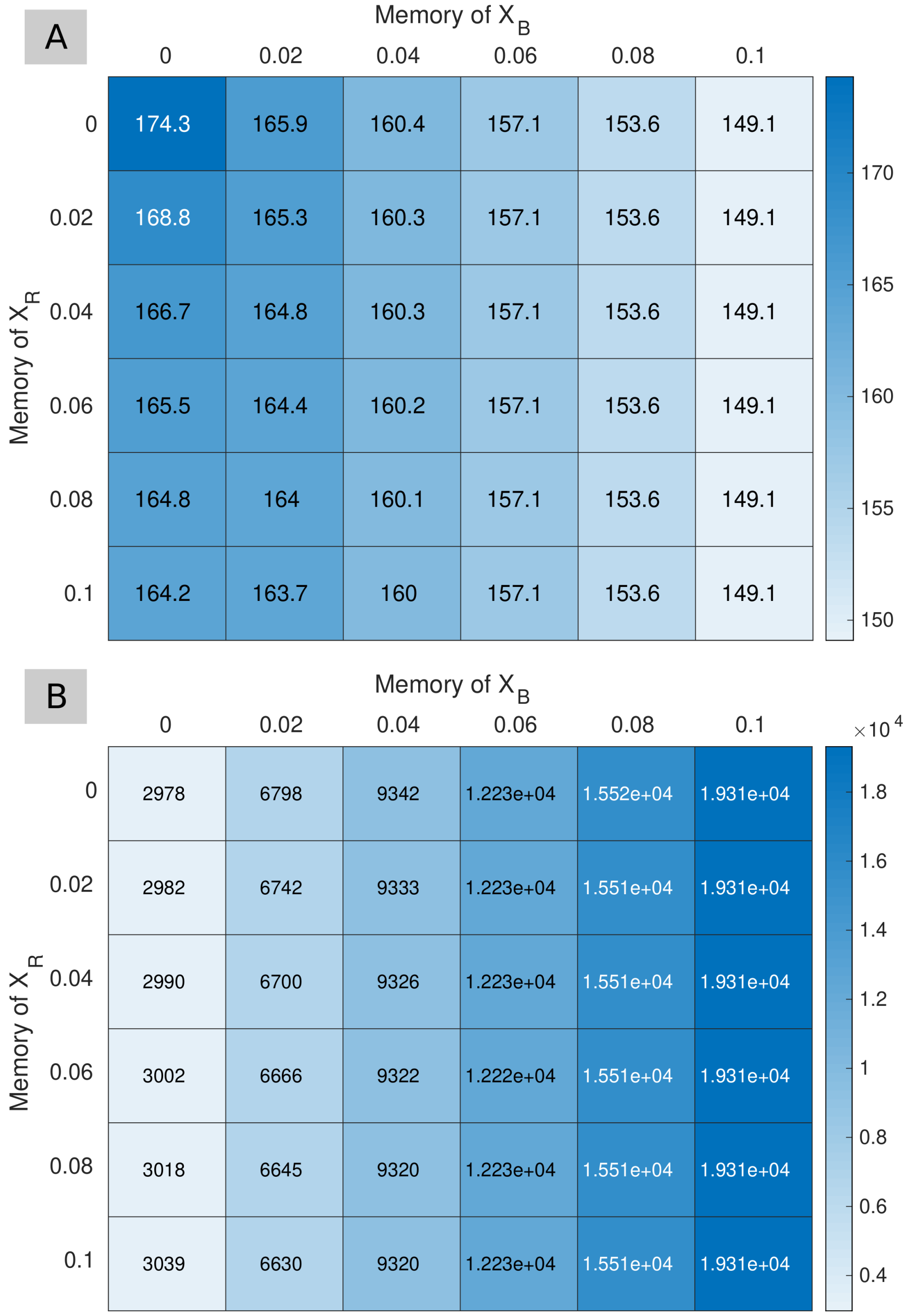

Fig S8. Impact of memory on resilience after a pulse perturbation in the two-species version of Gonze multistable model. Similar to Fig 7E-F. Both color and matrix entries indicate the recovery time after a pulse perturbation starting from the red-dominated stable state as a function of memory strength in the blue and red species (abundances X_B and X_R, respectively)). **(A)** Recovery times are first measured using a loose convergence interval of 0.02 on the Bray-Curtis dissimilarity of the community to its initial stable state, thus capturing the early stages of the recovery (see Resistance and resilience metrics section in Methods). Recovery time decreases with increasing memory, i.e., memory effects increase resilience. **(B)** Recovery times are then measured using a much tighter convergence interval of $1e-6$, thus capturing the later stages of the recovery. Recovery time now decreases with increasing memory, i.e., memory effects decrease resilience. Hence, the effect of memory on resilience depends on the time scale considered: memory hastens the recovery at first (A) but slows it down further in time (B).
