# Supplementary material for: Quantifying the impact of ecological memory on the dynamics of interacting communities
Source: PLoS Comput Biol. 2022 Jun 3;18(6):e1009396. doi: 10.1371/journal.pcbi.1009396 (PMC9200327; doi:10.1371/journal.pcbi.1009396)
Supplement: S9 Fig — (PDF) [file pcbi.1009396.s013.pdf]

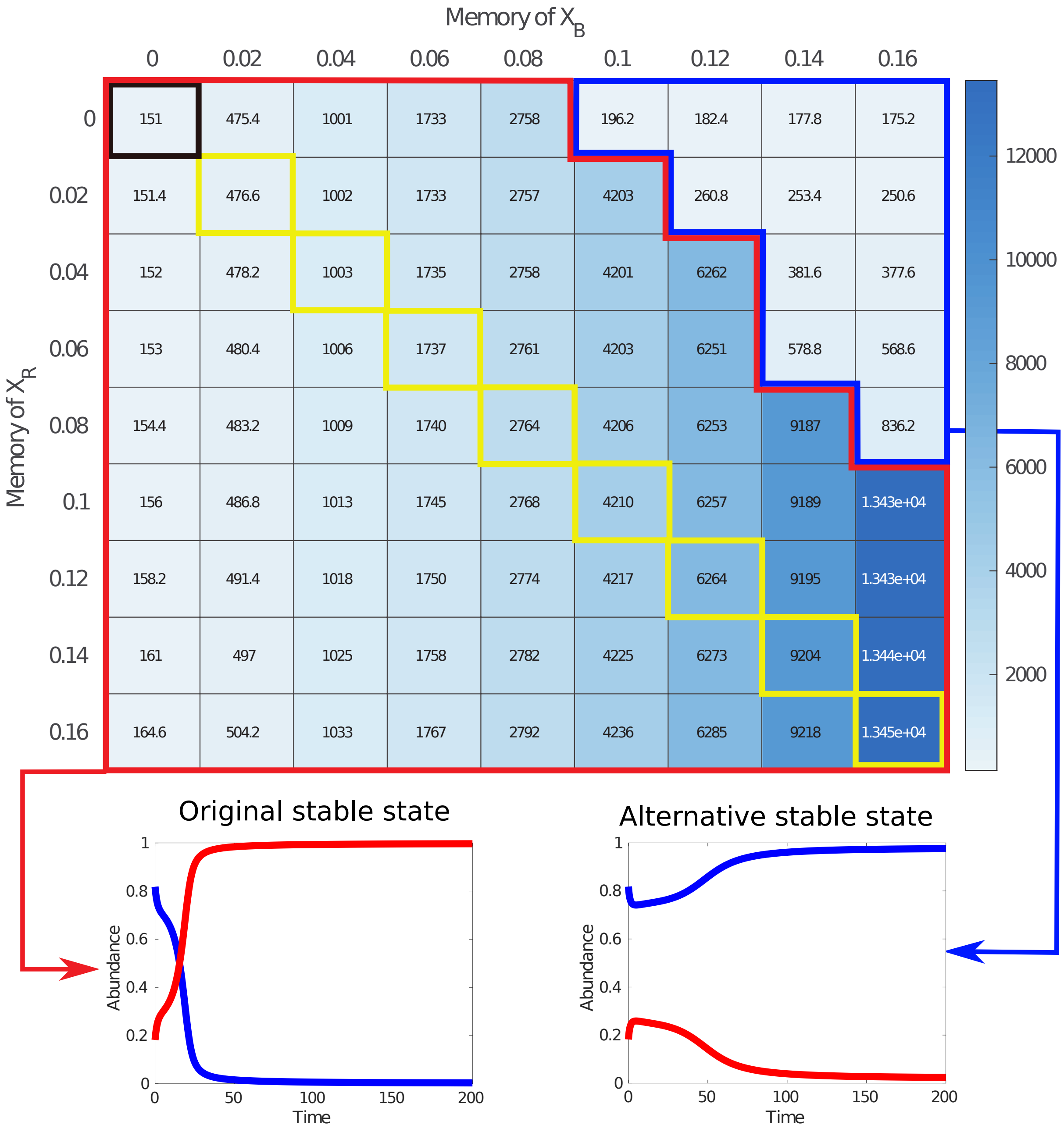

Fig S9. Impact of memory on convergence time in the two-species version of Gonze multistable model. Both color and matrix entries indicate the convergence time to stable state in the absence of perturbation as a function of memory strength in the blues and red species (abundances X_B and X_R, respectively). The upper-left cell corresponds the memoryless case (thick black border). Diagonal cells correspond to commensurate memory (yellow border). Cells with a red border correspond to communities that converge to the same stable state (dominated by the red species) as the community without memory, whereas cells with a blue border correspond to communities that converge to the alternative stable state (dominated by the blue species). In the red region of the matrix, increasing memory in either species increases the convergence time to the stable state (i.e., slows down the convergence). In the blue region, increasing memory in X_R also increases the convergence time, although increasing memory in X_B has little effect. On the other hand, increasing memory reduces the convergence time when it leads to a change in stable state.
