# Supplementary material for: Quantifying the impact of ecological memory on the dynamics of interacting communities
Source: PLoS Comput Biol. 2022 Jun 3;18(6):e1009396. doi: 10.1371/journal.pcbi.1009396 (PMC9200327; doi:10.1371/journal.pcbi.1009396)
Supplement: S10 Fig — (PDF) [file pcbi.1009396.s014.pdf]

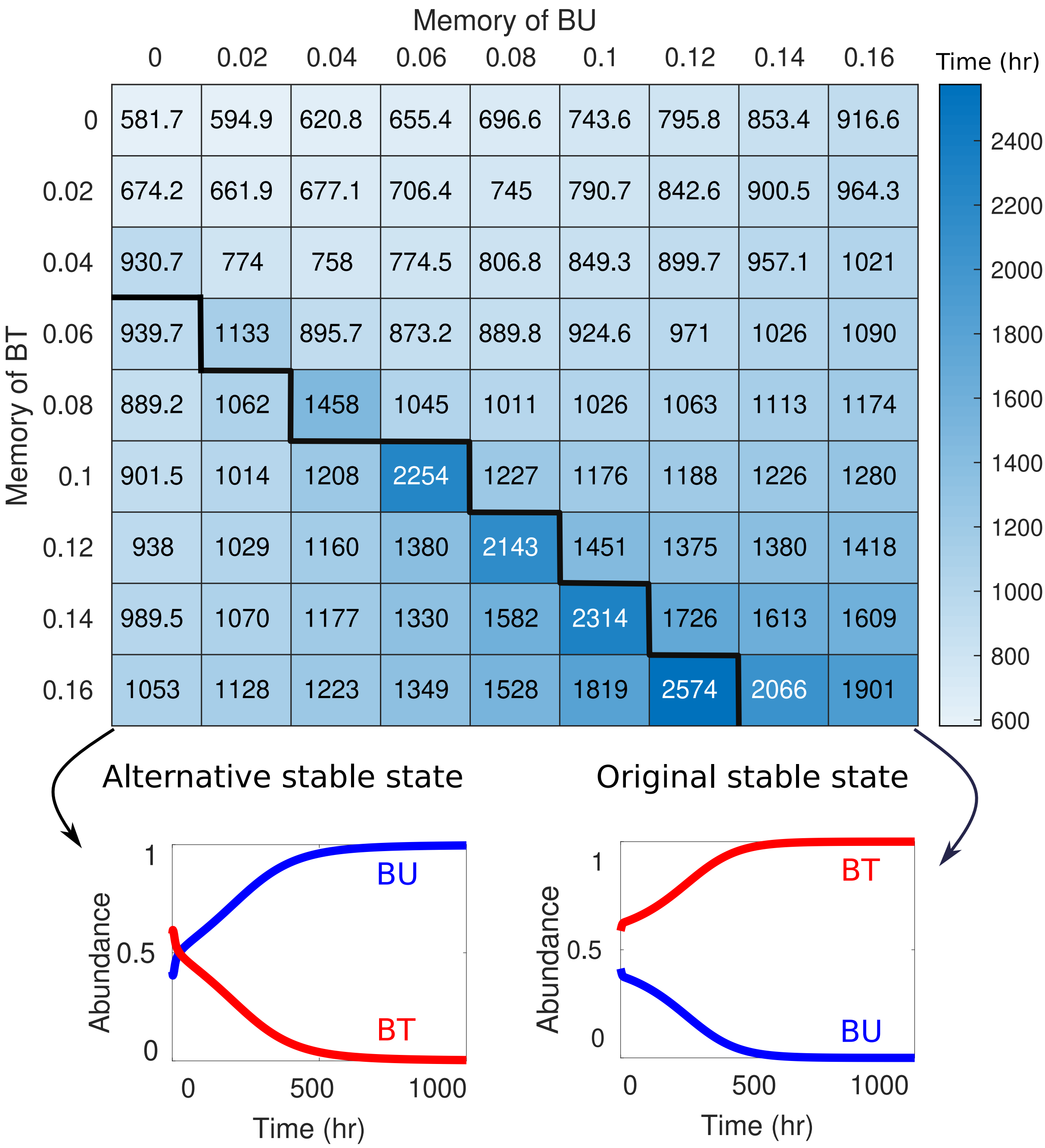

Fig S10. Impact of memory on convergence time in a two-species community exhibiting bistability between dominance of *Bacteroides uniformis* (BU) and *Bacteroides thetaiotaomicron* (BT). Both color and matrix entries indicate the convergence time to stable state as a function of memory strength in BU and BT. The upper-left cell corresponds to the memoryless case. Diagonal cells correspond to commensurate memory. Cells above the thick black border correspond to communities that converge to the same stable state (dominated by BU) as the community without memory, whereas cells below this border correspond communities that converge to the alternative stable state (dominated by BT). In the region of the matrix above the state transition, increasing memory in either species increases the convergence time to the stable state (i.e., slows down the convergence). In the region of the matrix below the state transition, increasing memory in BU also increases the convergence time, although increasing memory in BT has little effect. On the other hand, increasing memory may either increase or reduce the convergence time when it leads to a change in stable state.
