# Supplementary material for: Quantifying the impact of ecological memory on the dynamics of interacting communities
Source: PLoS Comput Biol. 2022 Jun 3;18(6):e1009396. doi: 10.1371/journal.pcbi.1009396 (PMC9200327; doi:10.1371/journal.pcbi.1009396)
Supplement: S11 Fig — (PDF) [file pcbi.1009396.s015.pdf]

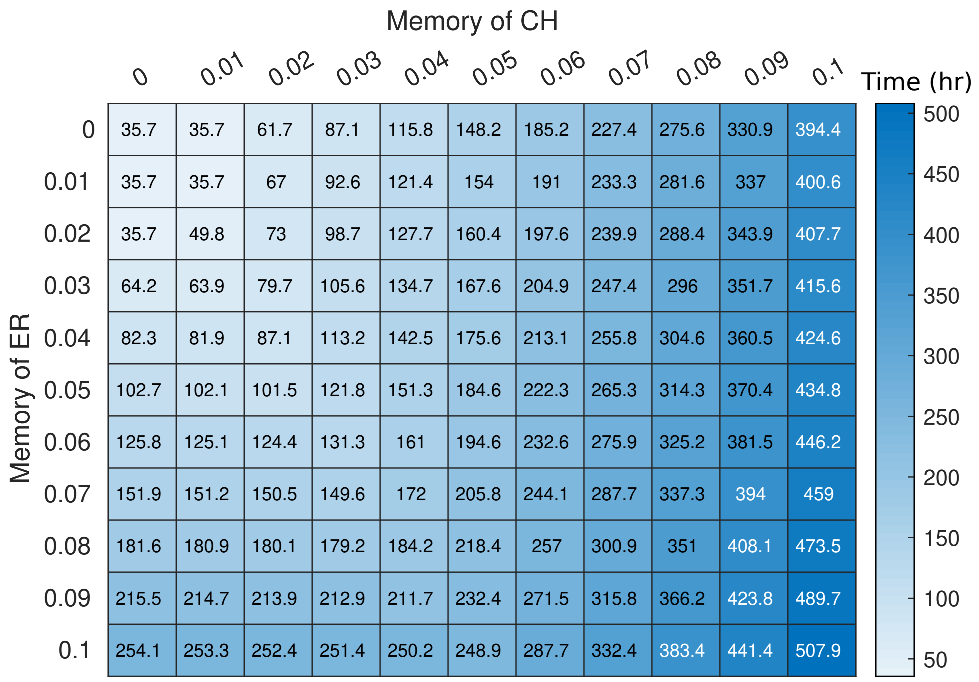

Fig S11. Impact of memory on convergence time in a two-species community exhibiting stable coexistence between *Eubacterium rectale* (ER) and *Clostridium hiranonis* (CH). Both color and matrix entries indicate the convergence time to stable state as a function of memory strength in ER and CH. The upper-left cell is the memoryless case, and the diagonal cells correspond to commensurate memory. Increasing memory in either species monotonically slows down the speed of convergence to the stable state.
