# Supplementary material for: Quantifying the impact of ecological memory on the dynamics of interacting communities
Source: PLoS Comput Biol. 2022 Jun 3;18(6):e1009396. doi: 10.1371/journal.pcbi.1009396 (PMC9200327; doi:10.1371/journal.pcbi.1009396)
Supplement: S12 Fig — (PDF) [file pcbi.1009396.s016.pdf]

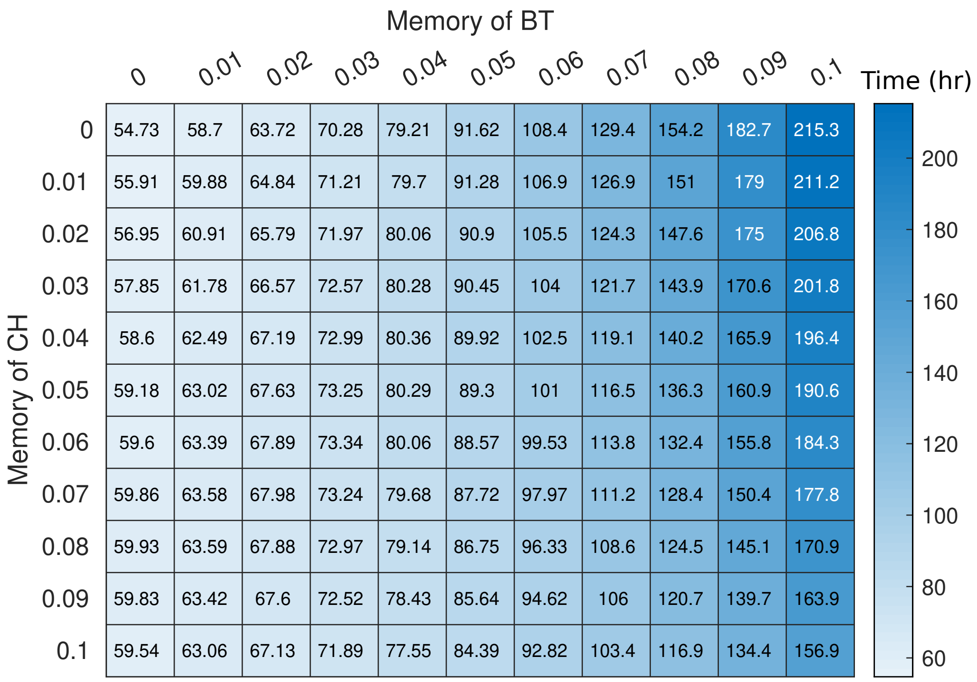

Fig S12. Impact of memory on convergence time in a two-species community exhibiting stable dominance of *Clostridium hiranonis* (CH) by *Bacteroides thetaiotaomicron* (BT). Both color and matrix entries indicate the convergence time to stable state as a function of memory strength in BT and CH. The upper-left cell is the memoryless case, and the diagonal cells correspond to commensurate memory. Increasing memory in either species monotonically slows down the speed of convergence to the stable state.
