# Supplementary material for: Quantifying the impact of ecological memory on the dynamics of interacting communities
Source: PLoS Comput Biol. 2022 Jun 3;18(6):e1009396. doi: 10.1371/journal.pcbi.1009396 (PMC9200327; doi:10.1371/journal.pcbi.1009396)
Supplement: Attachment — Submitted filename: Response to Reviews.pdf [file pcbi.1009396.s017.pdf]

Response to the reviewers' Comments:

Reviewer #1:
The authors study the impact of memory on the dynamics of generalized Lotka-Volterra systems. They show that memory can impact the resilience and resistance of dynamic systems after various perturbations and may impact the resulting stable states.

I think the premise of the manuscript is appealing and it is generally well presented. I agree with the authors that dynamic models can give quantitative insight into the behavior of microbial communities and their results support the general conclusion for the particular system and parametrization that was studied. The manuscript is a bit unclear on what biological system(s) the authors want to simulate which makes it hard to evaluate if the used parametrization has any connection to a real ecosystem or is mostly arbitrary.

Even though the manuscript is written excellently some parts are aimed at a very narrow audience and would benefit from some intuitive explanations to make it accessible to readers not familiar with fractional calculus (such as myself).

We thank the reviewer for these positive comments. We have now done our best to improve the manuscript in these respects; kindly find below our point-by-point responses. Line numbers refer to the manuscript without change tracking.

Suggested major changes

The use of fractional calculus to introduce memory into the generalized Lotka-Volterra model is probably the main contribution in the paper but very little intuition is provided on why that approach is better than alternatives mentioned in lines 21-25.

Our modeling approach based on fractional calculus has several advantages compared to the approaches mentioned in the introduction, and they are now more clearly laid out in the introduction (lines 23-32) and the discussion (lines 227-240). First, our approach allows describing long-term memory, with a power-law decay in the influence of past states on the present. Second, it allows the extension of existing ordinary differential equation models in a straightforward manner, by introducing non-integer derivative orders. The resulting fractional-order differential equation models can be solved in a computationally efficient way (we provide a Matlab code based on Fast Fourier Transform to this end). Finally, the fractional calculus framework benefits from abundant mathematical literature and well-established mathematical properties, such as the existence and uniqueness of solutions, and it has already been applied to model memory in other contexts (see https://doi.org/10.1016/j.cnsns.2018.04.019).

It is also hard to imagine what this particular memory effect actually looks like. So I would have liked to see a figure illustrating the shape of the fractional derivative under various values of μ. For instance, one could have shown the classic logistic growth curve along with varying fractional derivatives or a single impulse curve where one can observe how the impulse propagates in time due to memory. This would make the article accessible to readers without deep knowledge of fractional calculus and give an intuition on what time scales the modeled memory acts.

As suggested by the reviewer, we now illustrate the effect of various μ values on a logistic growth curve in the “Incorporating memory using fractional calculus” subsection of the

Methods, alongside an intuitive sketch of memory effects (Figure 7). Furthermore, we have illustrated the dynamics of four types of simple two-species communities (exhibiting either bistability, stable coexistence, or dominance) under various memory strengths (see Figure S5 and the next two responses).

The power-law shape of the kernel means that the memory we introduce acts across different times scales. This is illustrated by the impact of memory on resilience to perturbation: memory first hastens the return to the stable state at short time scales but delays it over longer time scales. We believe that the type of memory introduced by fractional derivatives can be considered as a general model for gradually declining long-term memory, as now stated in the discussion (229-235).

The authors keep the modeled systems very general which I think is fine. However, at least some part of the manuscript should be focused on a simulation of a system that was parameterized with experimental data to see whether the observed behavior extends to realistic settings. I think the works of Venturelli et. al. could be used here since they estimate gLV parameters in very controlled settings and with extensive data (see https://doi.org/10.15252/msb.20178157 or https://doi.org/10.1038/s41467-021-22938-y, a subset of taxa would be sufficient).

We thank the reviewer for this suggestion. We have followed it and incorporated memory effects into the gLV model proposed by Venturelli et. al. 2018. For demonstration purposes, we have formed three empirically parameterized two-species communities exhibiting different behaviors (bistability, stable coexistence, or dominance), and compared them to a two-species version of the Gonze model analyzed in the rest of the paper. We have added to the results an “Empirically parameterized model” subsection where we report the analysis of these models in the presence of memory. In this part, we also systematically vary the strength of memory in each model to assess the robustness of our findings.

In its current form, it is hard to evaluate whether the parameters of the models in Appendix S2 were chosen arbitrarily or selected to produce the particular results observed in the paper. An alternative would be to perform a robustness analysis on those parameters but I would consider this less preferable due to the large numbers of parameters and because behavior arising in only a small part of the parameter space may still be biologically relevant.

We have strived to clarify in the text where the parameters have been selected to illustrate a specific behavior, such as in Figure 4c and Figure 5d, and where parameters have been chosen more arbitrarily to illustrate general behaviors. In addition, we show in the new Figure 6 where the multistability and long instability regions are located in the parameter space of the model. Finally, the greater simplicity of the newly introduced two-species communities mentioned in the previous response allowed us to conduct a systematic assessment of memory effects in these communities, which we report as heatmaps in Figures S6 to S13. Figures S6 to S9 report their time of convergence to the stable state and Figures S10 to S13 their resistance and resilience to a pulse perturbation, as a function of memory strength in either species.

Suggested minor changes

It would be great if the authors could expand on which biological mechanism they consider

for memory. They mention some examples in lines 209-211. Different memory mechanisms may have vastly different time scales. For instance, acquiring a plasmid may give nearly unlimited memory that exceeds the doubling time but transcriptional regulation is very transient. It is likely that some of those mechanisms are captured better by fractional derivatives than others.

As mentioned above, the memory described by fractional derivatives is suitable for representing any phenomenon with a gradually decreasing long-term memory. Indeed the time dependency of such phenomena can often be well approximated by power-law functions, which are ubiquitous in nature. A recent paper describes for instance how the lag time of antibiotic-tolerant persister cells is distributed as a power-law function of time (https://www.pnas.org/content/116/36/17635; now cited in introduction and discussion, lines 28 and 211). More generally, we foresee that memory emerging at the population or community level from phenotypic heterogeneity would be likely to exhibit power-law distributed long-term memory, and hence to be well modeled by fractional derivatives, as now stated in the introduction and discussion (lines 26-29, 42-44, and 232-236). We propose dormancy, spatial structure, and adaptive bet-hedging as possible mechanisms leading to this type of memory at the community level, as now stated in the introduction and discussion (lines 17-20 and 212).

There are some formatting issues in the supplement. The table in Appendix S2 is not numbered and there is some overlapping text on line 492.

We have now fixed this and checked the manuscript carefully throughout.

I feel like Appendix S1 is pretty essential for what is happening in the manuscript, so I would probably convert it to a methods section in the main text (that may be located at the end of the manuscript).

We thank the reviewer for the suggestion, and we have now moved Appendix S1 to the Methods section as already mentioned.

I don’t have access to a Matlab license so I can not vouch that the provided source code works. I could imagine that providing some more information on how to install dependencies and in which order things should be executed to reproduce the results in the paper may be appreciated by potential readers. The Github repository linked to the Zenodo submission should be mentioned in the paper as well.

We thank the reviewer for the suggestion. Following the journal guidelines, we mention the Github repository, linked to a permanent Zenodo doi, in the Data availability statement at the end of the Methods (line 374). In the repository, we have included instructions for reproducing the results in the paper and expanding on them with arbitrary setting, as well as a link to our recently developed open-source Julia package for solving fractional derivative differential equations.

Reviewer #2:

This is an interesting and well written paper about under-explored topic about the role of "memory" in the community dynamics. I believe this is an important study. Unfortunately, I cannot comment on mathematical implementation of the models because I am not an expert in this field. Several general comments from more biological prospective regarding the manuscript are below.

We thank the reviewer for these positive comments. Please find below our point-by-point responses. Line numbers refer to the manuscript without change tracking.

Major comments.

1) The figure 5C provides a nice example of possible outcomes of community under memoryB=0.4. I believe for the broader audience it would be interesting to see several examples of the "space" of outcomes on a similar figure when all three species have incommensurate memory which is > 0 . (Unless this is computationally too difficult.)

We have systematically explored the outcome of the model presented in Figure 5c by varying memory strength across a range of values for all three groups of species. We report these results in Figure S2. We find that memory consistently reduces the average relative abundance of the group of species where it is introduced, in agreement with our conclusion in Figure 5c for a specific set of memory values.

2) How community assembly forces such as the ecological drift and selection relate to memory? Is it possible to speculate to what extent memory facilitates either drift or selection or both?

Memory is a systemic property that can arise from a wide range of mechanisms. In particular, it can emerge at the cellular level due to inertia in intracellular mechanisms, or it can emerge at the population or community level through phenotypic heterogeneity (such as in antibiotic resistant cell persistence). It can also be mediated by modifications of the extracellular environment by the organisms. Therefore, memory cannot be directly compared to fundamental ecological mechanisms such as drift and selection, however, both drift and selection may play a role in the emergence of memory at the community level. We have now expanded in the introduction and discussion on the biological mechanisms underlying memory and how they may relate to our modeling framework (lines 15-32 and 204-212).

In general, memory tends to dampen fluctuations, and can thus be expected to mitigate the effect of both drift and selection. In particular, we show in the Results that memory favors species coexistence in the presence of alternating pulse perturbations or stochastic perturbations (lines 90-91 and 116-119).

3) It probably goes beyond the scope of the paper but it would be very interesting to have at least one example of empirical estimates of the memory for a publicly available experimental/observational real or mock community timeseries.

We have now added simulations of three simple two-species communities using a model parameterized with empirical data from synthetic human gut bacterial communities, and we have systematically assessed the effect of memory on these communities. We report

these results in a new subsection of the Results entitled “Empirically parameterized model” (line 156).

Inferring memory from empirical data is indeed beyond the scope of this paper. Nevertheless, we now point out in the discussion that fractional-order differential equation models can be efficiently solved numerically, and might therefore be amenable to simulation-based inference, which would represent a promising avenue for future research (lines 239-240).

4) Would the interpretations of outcomes of the model be applicable for absolute abundances?

We actually model absolute abundances throughout the paper. We report relative abundances in some figures only to make representation and comparison easier. We have now clarified this point in Methods (lines 302-304).

5) It would be useful to have a table or graphical summary in what instances what combinations of values of memory will lead to resistance and when to resilience and recovery of pre-disturbance state.

We now analyze four simple two-species communities, including three communities parameterized with empirical data as already mentioned, and a two-species version of the model studied in the rest of the paper for comparison. The reduced number of parameters in these communities allowed us to systematically assess the effect of memory on the time to convergence, resistance and resilience by varying the strength of memory in the two species. We introduced to this end quantitative measures of resistance and resilience (see Resistance and resilience metrics, line 349). We report the results in the new “Empirically parameterized model” subsection of the Results (line 156), and graphically as heatmaps in Figures S6 to S13.

Minor comments.
1) Statement in the abstract that memory “..thus reducing the system's resilience” seems too strong since later figure 2 shows actually that memory>0 appears to promote resilience.

Thank you for pointing this out, we have now replaced “reducing” by “affecting”.

2) Page 25. Line 510. Fig.3?. Figure number missing

This is now fixed, thank you.

3) Page 24. After line 505. Table number is missing, I assume it is referred to as Appendix S2

This is now fixed, thank you.

4) Double-check if used shades of the green/red colors are color-blind friendly.

This is indeed an important consideration, and we have used an online color-blindness simulation tool to ensure that all colors used in the figures are color-blind friendly

(https://davidmathlogic.com/colorblind/#%23D81B60-%231E88E5-%23FFC107-%23004D40).

Reviewer #3:

In the manuscript of “Quantifying the impact of ecological memory on the dynamics of interacting communities”, the authors use the framework of fractional calculus to study how the outcomes of a well-characterized interaction model are affected by gradual increases in ecological memory under varying initial conditions, perturbations, and stochasticity. Results highlight the implications of memory on several key aspects of community dynamics.

The research background is of great interest. The manuscript is well written. However, I have several questions especially on techniques, for example, the quantity of stability, and so on.

We thank the reviewer for the positive comments. Please find below our point-by-point responses. Line numbers refer to the manuscript without change tracking.

Major questions:

1) As shown in SM, the authors started from the memoryless model and then incorporated memory. Could the authors explain the generality of the integral function, Eq. (s) in SM, denoting the effects of memory. Looks like that this definition is crucial. Without different definitions, results could be totally different.

From a mathematical viewpoint, the shape of the kernel is dictated by the definition of fractional-order derivatives. They are a natural extension of ordinary integer-order derivatives that has been extensively studied and that exhibits desirable mathematical properties, for instance regarding the existence and unicity of solutions for the resulting differential equations. This kernel shape, therefore, allows readily introducing memory in existing ordinary differential equation models by simply replacing ordinary with fractional-order derivatives, which would not necessarily be possible for other kernel shapes. The resulting differential equations can also be readily solved numerically. This is now more clearly explained in the Discussion (lines 227-240).

From a biological viewpoint, the power-law shape of the kernel introduced by fractional-order derivatives can be regarded as a fairly general way to model gradually declining long-term memory. Indeed the time dependency of such phenomena can often be well approximated by power-law functions, which are ubiquitous in nature. A recent paper describes for instance how the lag time of antibiotic-tolerant persister cells is distributed as a power-law function of time (https://www.pnas.org/content/116/36/17635; now cited in introduction and discussion, lines 28 and 211). More generally, we foresee that memory emerging at the population or community level from phenotypic heterogeneity would be likely to exhibit power-law distributed long-term memory, and hence to be well modeled by fractional derivatives, as now stated in the introduction and discussion (lines 26-29, 42-44 and 232-236).

2) Based on the constructed model, there are also different ways of perturbations, as mentioned in Fig. 1, including Pulse, periodic, stochastic, and so on. Could the authors

quantify the effects of parameter values of these different types of perturbations on the stability, recovery time, and resilience.

We fully agree about the importance of checking the robustness of our results to variations in parameter values. Consequently, and following the suggestions of other reviewers, we have conducted a systematic analysis of the effect of memory on convergence time, resistance, and resilience over a wide range of memory strength values. We report the results in the new “Empirically parameterized model” subsection of the Results (line 156), and graphically as heatmaps in Figures S6 to S13.

Variations in perturbation parameters are in comparison less essential to our results since our work focuses on the effect of memory on the system. We found that our results are robust overall to the exact shape and strength of the perturbations, except around certain threshold values that separate qualitatively different behaviors. In those cases, we have strived to illustrate values of perturbation parameters on both sides of the thresholds (e.g., the intensity of a pulse perturbation leading to a shift in stable state in Fig. 2). Furthermore, our new systematic assessment of resistance in two-species bistable communities consists in determining for each memory value the strongest pulse perturbation for which the community recovers to its initial stable state (Fig. S10 and S11).

3) Could the authors provide the definition of stability, recovery time, resilience?

We have now included a new subsection in the Methods, “Resistance and resilience metrics”, which introduces quantitative metrics for resistance and resilience (line 349). We used these metrics explicitly in our new analysis of the effect of memory on convergence time, resistance, and resilience (line 156, Fig. S6 to S13).

As mentioned above, we quantified resistance as the strongest pulse perturbation for which the community recovers to its initial stable state. We quantified resilience as the time needed for a community to recover to its initial stable state after a perturbation. The recovery is defined up to a certain threshold, and setting a more or less stringent threshold allows emphasizing longer-term or shorter-term dynamics, respectively. Finally, all the dynamical systems studied in this work are stable, which is to say that the dynamics always converge to a stable state eventually (although there may be several, and to which of them the dynamics converge will then depend on initial conditions). Therefore, we did not use a metric for stability.

So, in general, the research question is very interesting. But it would be nice to provide the definition of metrics clearly and investigate the effects of memory rigorously.

Thank you for this positive comment. As already mentioned, we have now included a systematic analysis of the effect of memory on convergence time, resistance, and resilience in four simple two-species communities exhibiting a range of different behaviors (multistability, stable coexistence, dominance; line 156, Fig. S6 to S13). We have used in these analyses quantitative metrics for convergence time, resistance, and resilience (line 346).
