# Supplementary material for: Quantifying the impact of ecological memory on the dynamics of interacting communities
Source: PLoS Comput Biol. 2022 Jun 3;18(6):e1009396. doi: 10.1371/journal.pcbi.1009396 (PMC9200327; doi:10.1371/journal.pcbi.1009396)
Supplement: Attachment — Submitted filename: Response to the reviewers Comments_.pdf [file pcbi.1009396.s018.pdf]

**Response to the reviewers' Comments:**

**Reviewer #1:**

The authors have expanded their explanation of what phenomena could give rise to the modeled memory effects and now provide a much better illustration and description of how fractional calculus can be used to model the covered memory effects. Additionally, the manuscript now also includes a small study of memory effects within a gLV model derived from bacterial growth curves, thus, providing simulations of memory effects in a somewhat more realistic setting. I think this has already improved the manuscript

We thank the reviewer for these positive comments.

and I only have some minor suggestions to improve clarity and presentation of the data.

We have now improved the manuscript in these respects; kindly find below our point-by-point responses. Line numbers refer to the manuscript with change tracking.

Minor suggestions

The manuscript text and methods are both pretty unclear on how the data from Venturelli et. al. was integrated. Did the authors use the parameters fit in the original manuscript or was the fractional calculus model refit to the time series data?

We have now clarified in both the main text and the Methods that we indeed used the parameters fitted by Venturelli et al., and then introduced memory in the corresponding model:

- "To study whether the observed memory effects also arise in a more realistic setting, we applied our approach to a gLV system parameterized by Venturelli et al. [39] with experimental data from synthetic human gut microbial communities." (Lines 158-161).
- "We considered four microbial species, based on the values inferred in [39] for their interaction coefficients and growth rates (see Appendix Figure S27 in [39])." (Lines 334-335).

In my opinion this data should have been a figure in the main manuscript as I find simulations in more realistic settings much more interesting than some of the more artificial settings in the other figures. The growth curves shown in Fig. S5 should also be presented alongside with the actual growth curves from the Venturelli manuscript to give an impression how well the simulations match the measured growth curves.

Thanks for the suggestion. We are now presenting as a main figure (Fig 7) results related to the bistable community obtained by combining *Bacteroides uniformis* (BU) and *Bacteroides thetaiotaomicron* (BT). In both this figure and in S5 Fig, we have plotted the original data points from Venturelli *et al*. alongside the simulated growth curves. We simulated the growth curves using the parameters fitted in Venturelli *et al.* and starting from the same initial conditions.

Line 158: I don’t think what was done here constitutes an actual validation since they never compare the simulated memory behavior to measured data. Rather it should state something along the lines of: “To study whether the observed memory effects may arise in a more realistic setting…”, which is what was actually done here.

We have now modified this sentence as suggested by the reviewer: “To study whether the observed memory effects also arise in a more realistic setting …” (line 158), and removed “validate” throughout (Author summary and lines 47 and 329).

The heatmaps in figures S5-S8 and others are missing units for the color bars. Is the recovery time expressed in hours, minutes, or seconds?

We thank the reviewer for pointing this out. We now specify in the figures that the recovery times are in hours.

I think the points brought up by the other reviewer are quite important. If the modeling strategy can not account for selection and drift this should be stated clearly in the manuscript.

Our approach to modeling memory using fractional derivatives is phenomenological: it does not assume a specific ecological mechanism for the emergence of memory, but aims at introducing gradually declining long-term memory effects in a general way. As such, it can be combined with many different types of ecological models, including models that account more specifically for ecological mechanisms such as selection and drift. This has now been clarified in the Discussion (lines 238-240 and 245).

Nevertheless, we mainly demonstrate our modeling approach on a specific model introduced by Gonze *et al.* (2017), as well as on related generalized Lotka-Volterra models. These deterministic models focus on biotic interactions between bacterial species and do not explicitly take into account selection by the environment and stochastic drift in species abundances. However, they could easily be extended to take into account these mechanisms. This has now also been clarified in the Discussion (lines 228-231).

**Reviewer #2:**
Authors have addressed all of my questions and suggestions.

We thank the reviewer for the positive comment.

In caption to Figure S4, reference to another figure is missing and has two question marks "see Fig. ??"

Thanks for the kind reminder. We have now fixed it and checked the manuscript carefully throughout.
